# Appointment of Dr. Billings

**Published:** 1885-04

**Authors:** 


### THE APPOINTMENT OF DR. BILLINGS.

It will be, no doubt, a source of pleasure and satisfaction to our readers to learn of the appointment of our associate, Dr. Billings, to the chair of Pathology and Patho-mycology at the Polyclinic Medical College of this city. The event is an important one in the history of our profession, as heretofore no member of it has held the position of instructor in any human medical school in this country or in England.

Every thoughtful veterinarian cannot fail to see in it an opening wedge which will obtain entrance for him into the ranks of his hitherto exclusive fellow-workers, and that it dates a new era in the history of medicine, which holds out to his profession wider fields of usefulness and honorable distinction.

The Medical College which has inaugurated this new departure in the onward march of Medical Science has reason to congratulate itself on the step it has taken. Comparative medicine is destined to exert a powerful influence for good over her sister science, recalling her from the narrow grooves into which she has run, and placing before her broad pathways which invite to profitable investigation.

We wish Professor Billings success in his new undertaking. C.

